# Microsporidia and Its Relation to Crohn's Disease. A Retrospective Study

**DOI:** 10.1371/journal.pone.0062107

**Published:** 2013-04-18

**Authors:** Juan C. Andreu-Ballester, Carlos Garcia-Ballesteros, Victoria Amigo, Ferran Ballester, Rafael Gil-Borrás, Ignacio Catalán-Serra, Angela Magnet, Soledad Fenoy, Carmen del Aguila, Jose Ferrando-Marco, Carmen Cuéllar

**Affiliations:** 1 Research Department, Arnau de Vilanova Hospital, Valencia, Spain; 2 Hematology Department, Arnau de Vilanova Hospital, Valencia, Spain; 3 Center of Research in Public Health, Valencia, Spanish Consortium for Research in Epidemiology and Public Health, Barcelona, University of Valencia, Valencia, Spain; 4 Digestive Department, Arnau de Vilanova Hospital, Valencia, Spain; 5 Laboratory of Parasitology, San Pablo Centro de Estudios Universitarios University, Madrid, Spain; 6 Anatomical Pathology Department, Arnau de Vilanova Hospital, Valencia, Spain; 7 Department of Parasitology, Faculty of Pharmacy, Complutense University, Madrid, Spain; Northwestern University Feinberg School of Medicine, United States of America

## Abstract

**Background:**

The cause of Crohn's Disease (CD) remains unknown. Recently a decrease in the global lymphocyte population in the peripheral blood of CD patients has been reported. This decrease was more evident in γδ T lymphocytes, especially γδ CD8+T subsets. Furthermore, a decrease of IL-7 was also observed in these patients. We propose the hypothesis that microsporidia, an obligate intracellular opportunistic parasite recently related to fungi, in CD patients can take advantage of the lymphocytes and IL-7 deficits to proliferate and to contribute to the pathophysiology of this disease.

**Methods and Findings:**

In this case-control study, serum samples were collected from 36 CD patients and from 36 healthy individuals (controls), IgE and IgG anti-*Encephalitozoon* antibodies were determined by ELISA; and forty-four intestinal tissue samples were analyzed through real time Polymerase Chain Reaction (PCR), twenty CD patients, nine with others diseases and 15 healthy subjects.

We observed that IgE anti-*Encephalitozoon* levels were significantly higher in patients with CD: 0.386(±0.256) *vs* control group, 0.201(±0.147), *P*<0.001. However, IgG anti-*Encephalitozoon* values were significantly lower in CD patients: 0.361(±0.256) *vs* control group, 0.876(±0.380), *P*<0.001. In the group of CD patients, 6/20 (30%) were positive by real time PCR for microsporidia and, all the patients of the control group were negative by real time PCR.

**Conclusions:**

These results suggest that CD patients are a group at risk for microsporidiasis and, moreover that microsporidia may be involved as a possible etiologic factor of CD.

## Introduction

The cause of Crohńs disease (CD) remains unknown. Much of the research on CD pathogenesis has been focused on immunology and especially on the role of lymphocytes in this disease. We have recently reported a decrease in the global lymphocyte population in the peripheral blood of patients with CD. This decrease was more evident in γδ T lymphocytes, especially γδ CD8+T subsets [1]. Furthermore, we also demonstrated in this group of patients a decrease of IL-7 [2].

Microsporidia are obligate intracellular opportunistic parasites capable of infecting a wide range of vertebrate and invertebrate hosts [3]; they were previously considered protozoa and recently they have been related to fungi [4], [5]. Microsporidia cause opportunistic infections in immunocompromised individuals, mainly HIV/AIDS patients [6]–[8]. However, the number of non-HIV-patients with other forms of immunosuppression is also increasing. Among these, organ transplant recipients have been considered as a group of patients at risk [9], [10]. On the other hand, cases in HIV-negative people, including travellers and elderly people, are increasing [11], [12] and it has been recently suggested that the incidence of microsporidial infections in healthy population is much higher than previously reported [13]–[15]. These opportunistic pathogens can cause a variety of systemic and non systemic manifestations; chronic diarrhea is the most common one [3].

Cellular immunity is known to be essential for the resolution of microsporidia infection. Studies on BALB/c mice demonstrated that resistance to lethal disease produced by *Encephalitozoon cuniculi* was T cell-dependent [16]. For protection, CD8^+^ T cells are more important, as knockout mice lacking CD8^+^ T cells were highly susceptible to *E. cuniculi* infection, whereas those lacking CD4^+^ cells were not [17]. Likewise, oral *E. cuniculi* infection produced a rapid increase of the intraepitelial lymphocyte (IEL) population in animals. These IEL populations were principally of the CD8 αα subset [18]. Studies carried out in mice have shown an early increase of γδ T cells during infection with *E. cuniculi* [19]. Interestingly, 50–70% of IEL of the intestinal mucosa are γδ T cells that express the CD8 molecule [20], [21].

Spores are the infective forms of these parasites, which are resistant to the environment. They contain a coiled polar tube, also called polar filament, that ejects under appropriate conditions (change in pH or osmotic pressure) and inject the spore cytoplasm through the polar filament into the host cell [3]. This fact distinguishes microsporidia from other unicellular spore forming organisms. Furuya et al [22] established three mouse monoclonal antibodies against *E. cuniculi* polar tube protein 1 (PTP 1) and all of them were IgE class, suggesting that this antigen may have the potential to mainly induce specific IgE antibody production.

There are no prior studies published that relate microsporidia to CD. We proposed the hypothesis that microsporidia could take advantage of the deficit of lymphocytes and IL-7 in patients with CD to proliferate and contribute to the pathophysiology of this disease.

On the other hand, there are no studies that specifically investigate if CD patients, due to their impaired cellular immunity, may be a risk group for microsporidia colonization. For this reason we have investigated microsporidia seroprevalence in a group of CD patients and the presence of these parasites in their tissues.

## Methods

### Study Population

In this retrospective study we used the same population recruited in a previous work [1]. We collected serum samples from 36 Crohńs disease patients and from 36 healthy individuals (controls). Serum samples were maintained at −80°C until analytical determinations were done. The 36 CD patients were selected following Lennard-Jones criteria for CD. Both groups were paired by sex and age±5 years. CD patients were divided according to three clinical scenarios: “new patients” with active CD presenting at, or shortly after, diagnosis with no previous treatment for CD, “remission” (CDAI<150 for at least 12 months) and “active disease” (CDAI >150and signs and symptoms of disease). The activity of the disease was evaluated according to Crohńs disease activity index (CDAI). Therefore, the group of CD patients was constituted by 13 (36.1%) new patients, 13 patients in remission (36.1%), and 10 patients with active disease (27.8%). Patients in remission were recruited among patients in follow-up at the outpatient clinic. On the other hand, new patients and patients with active disease were selected among patients admitted to the Gastroenterology Department at the Arnau de Vilanova Hospital (Valencia, Spain).

Healthy controls inclusion criteria were: absence of acute infections, inflammatory, autoimmune or immunodeficiency diseases; and no immunosuppressive or antibiotic treatment or any kind of vaccine during the previous year.

To study the presence of microsporidia, forty-four intestinal tissue samples were analyzed by PCR of which 20 samples correspond to CD patients, nine to patients with other intestinal diseases and 15 to healthy subjects that presented a normal exploration and no pathology after rectal endoscopy.

Each participant in the study signed an informed consent form, and the study was approved by the Ethics and Investigation Committee of the Arnau de Vilanova Hospital (Valencia, Spain).

### Variables Studied

The following variables were recorded: age and gender; Crohńs disease activity index (CDAI); Clinical Scenarios: remission, active disease, new patient; Complete blood count and αβ and γδ T cells subsets; IgE and IgG anti-*Encephalitozoon* antibodies and presence of microsporidia in tissue.

### Methods of Blood Sample Analysis

Blood cell counts were performed using Coulter LH750 automated haematology analyzer (Beckman Coulter, Fullerton, CA). Monoclonal antibodies used: CD45, CD4, CD8. CD3, CD19 for the peripheral blood subpopulations and CD4, CD8, CD56, CD2, CD3, CD19, TCRαβ y TCRγδ for the T γδ lymphocytes study. γδ T lymphocyte populations were analyzed with Phycoerythrin-Cyanine 5.1 (PC5) conjugated anti-human TCR γ-δ (clone: IMMU 510) (Beckman Coulter, Miami, FL). αβ T lymphocytes were analyzed with Phycoerythrin-Cyanine 5.1 (PC5) conjugated anti-human TCR α-β (Clone: IP26A) (Beckman Coulter, Miami, FL).

### *Encephalitozoon cuniculi* antigen and determination of specific antibodies

The *E. cuniculi* antigen was obtained from *E. cuniculi* (USP-A1) spores cultured on E6 monolayer following Aguila et al protocol [23]. Briefly, spores were disrupted using glass beads, 2.5% SDS and 2% mercaptoethanol. Soluble antigens were obtained from the supernatant after centrifugation. Protein content was determined by the Bradford method and adjusted to 0.8 µg/ml to coat ELISA plates. Duplicate dilutions of human sera at 1/200 in PBS-Tween, containing 0.1% BSA were added and incubated. Horse radish peroxidase (HRP) conjugate goat anti-human IgG (Biosource International, Camarillo, CA) was used. For IgE determination, test sera were added in duplicate at a 1/2 dilution. A murine monoclonal antibody against a human IgE chain (IgG1κ, E21A11, INGENASA, Madrid, Spain) was added and incubated, followed by a goat anti-mouse IgG1 (gamma) HRP conjugate (CALTAG Laboratories, Burlingame, CA) [24], [25].

### IL-7 determination

IL-7 Instant ELISA for human interleukin-7 (eBioscience) was used to measure IL-7 following manufacturer's instructions.

### Tissue sampling

All patients underwent the same standard bowel preparation prescribed in our hospital. Three to five biopsies of the ileum and colon were taken at 1–2 cm intervals in each patient. All samples were fixed through immersion in a solution of 10% buffered formalin. Specimens received in the histopathology laboratory were routinely processed by paraffin embedding. Tissue was stained by Hematoxylin-eosin and by Modified Trichrome stain [29].

### Polymerase Chain Reaction (PCR)

DNA extraction: DNA was extracted from two to four slices of paraffin embedded tissue of 3 µm thick. Deparaffination of sections was done with two xylene washes of 2 ml each followed by two ethanol washes of 2 ml each. Samples were dried at 40°C. DNA extraction was done with DNeasy® Blood & Tissue Kit (QIAGEN, La Joya, CA) following manufacturer's instructions.

Microsporidia PCR: a Syber green real time PCR described by Polley el al [26] was followed to detect the most common human microsporidia pathogens. Species identification was based on the melting temperature (Tm) of the amplicons. Positive controls for *E. bieneusi*, *E. hellem/intestinalis* and *E. cuniculi* were used.

Interpretation of results: for species identification the Tm of the sample was the positive control±0.1°C.

### Statistical Analysis

When normality was fitted, Student *t* test for paired samples was used to compare the means of the quantitative variables. Wilcoxon non-parametric test was used to study the variables that did not have a normal distribution. When we analyzed the differences between cases and controls according to the clinical categories, non-parametric Wilcoxon test was always used, because of the number of patients in each group. Correlation studies using Pearson's correlation coefficient were performed to compare immunoglobulin levels and lymphocytes subsets. The level of significance was taken as a *P* value of less than 0.05 (bilateral contrast). Data were analyzed using the statistical software SPSS, version 18.

## Results

### Anti-*Encephalizotoon* antibodies

IgE anti-*Encephalitozoon* levels were higher in the group of patients with CD when compared to the controls. Contrarily, IgG anti-*Encephalitozoon* values were lower in the CD group with respect to the control group.

**Figure 1:**
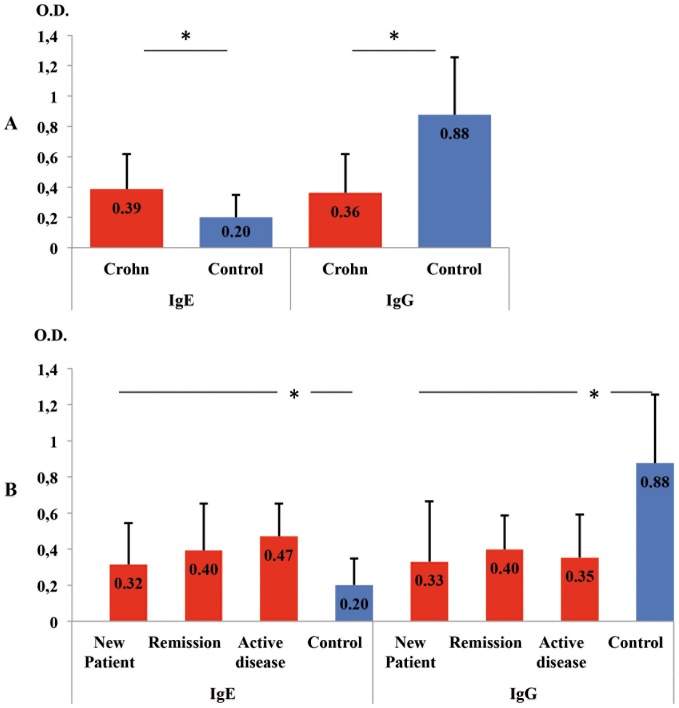
Anti-*Encephalitozon* antibody levels measured by ELISA (IgE and IgG). A: Crohn`s disease (N = 36) *vs* Control group (N = 36). B: Clinical scenarios of Crohn's disease, New patient (N = 13), Remission (N = 13), and Active disease (N = 10) *vs* Control group. Immunoglobulins are expressed in means of Optical Density (O.D.) values and T bars denote standard deviation. * *P*<0.001.

As it is shown in Figure 1A, we observed that IgE anti-*Encephalitozoon* levels were significantly higher in patients with CD, 0.386(±0.256) *vs* control group, 0.201(±0.147), *P*<0.001. However, IgG anti-*Encephalitozoon* values were significantly lower in CD patients, 0.361(±0.256) *vs* control group, 0.876(±0.380), *P*<0.001.

In Fig. 1B both immunoglobulin levels are described according to the different clinical scenarios that were studied in CD. There were significant differences (*P*<0.001) between the control group and the three clinical scenarios analyzed.

### Correlations among IgG and IgE anti-*Encephalitozoon* levels with lymphocyte subsets

**Figure 2:**
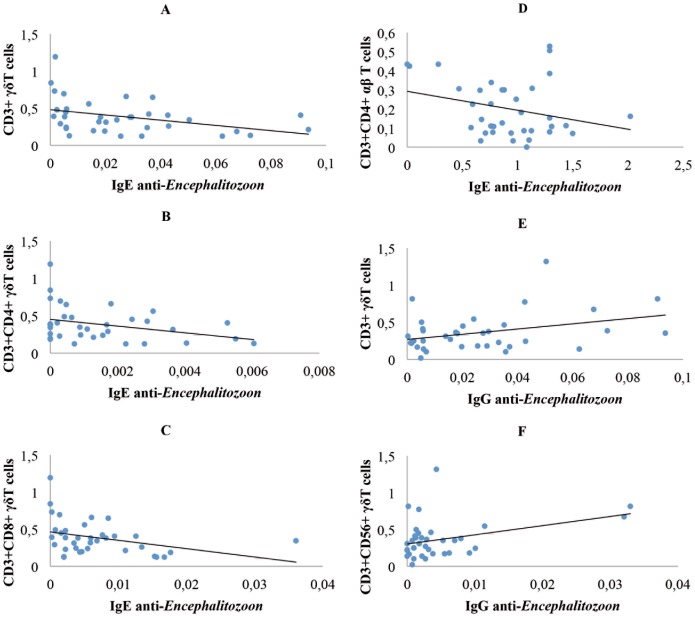
Correlation between immunoglobulins (IgG, IgE) anti-*Encephalitozon* and T cells subsets. A, B, and C express, in CD patients, the correlation between IgE anti-*Encephalitozoon* and CD3+γδ T cells (−386, *P* = 0.02) and CD3+CD4+γδ T cells (−338, P = 0.047), and CD3+CD8+γδ T cells (−350, *P* = 0.037), respectively. D, healthy subjets, describes the correlation of IgE anti-*Encephalitozoon* with CD3+CD4+αβ T cells (−540, *P*<0.001). E and F represent the correlations obtained in CD patients between IgG anti-*Encephalitozoon* antibody levels and γδ T cells (+346, P = 0.039) and CD3+CD56+γδ T cells (+362, *P* = 0.03), respectively. Immunoglobulines are expressed as means of Optical Density (O.D.). T cell values are expressed as means (x 10^9^/L).

As it is shown in the Figure 2, IgE anti-*Encephalitozoon* was negatively correlated with γδ T cells when the group of CD patients was analyzed. This correlation was dependent on CD4+ and CD8+γδ T cells.

IgE anti-*Encephalitozoon* was negatively correlated with αβ T cells when the control group was analyzed. This correlation was dependent on CD4+αβ T cells.

In the group of CD patients IgG anti-*Encephalitozoon* antibodies were positively correlated with γδ T cells. This correlation was dependent on CD56+γδ T cells.

**Figure 3:**
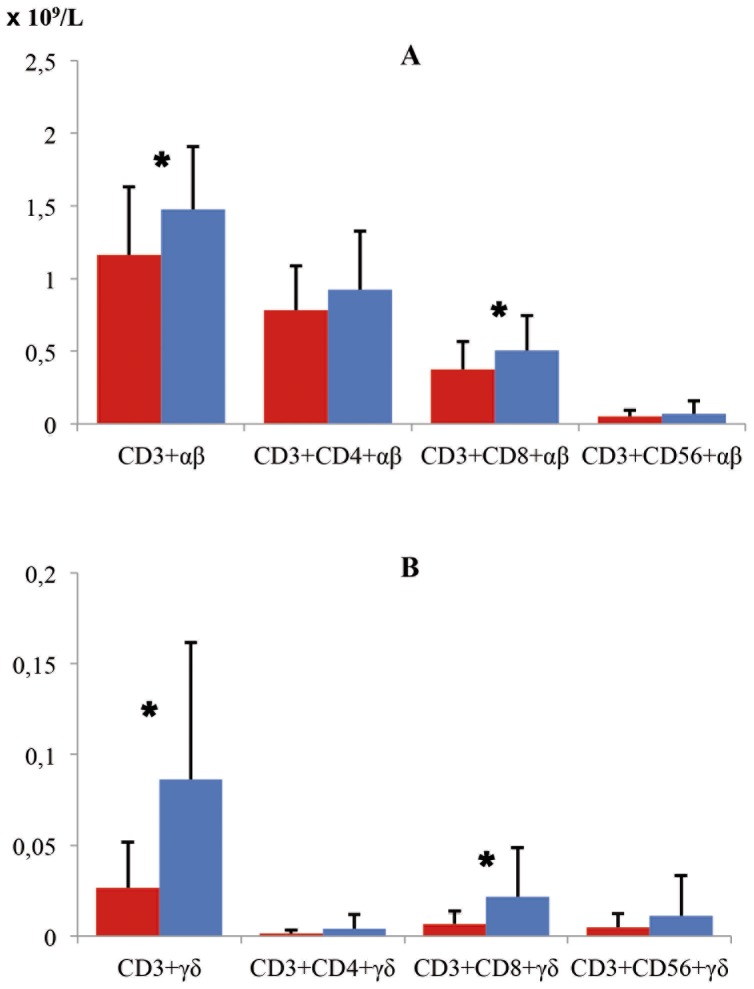
Values of T lymphocyte subsets according to type of receptor αβ (A) and γδ (B) in CD patients and Control Group. CD patients (red bars) and control groups (blue bars). Significant differences (*) were observed in the case of CD3+αβ (*P* = 0.006), CD3+CD8+αβ (*P* = 0.018), CD3+γδ (*P*<0.001) and CD3+CD8+γδ (*P* = 0.003). The deficit of T lymphocytes in patients with CD with respect to the control group was more intense when γδ T cells were studied.

In Figure 3 values of T lymphocyte subsets are shown according the type of receptor that they are expressing in their membranes [αβ (A) and γδ (B)].

### Relations among positive anti-*Encephalitozoon* IgE antibodies in CD patients and γδ T cell subsets

We analyzed the frequency of γδ T cells in CD patients related to positive anti-*Encephalitozoon* IgE antibodies. (Values higher than the mean of the Optical Densities of the 72 serum samples plus once their standard deviation were considered as IgE positive). We found a significant decreased of CD3+γδ T cells in IgE positive CD patients (N = 18) 0.0196±0.0228 *vs* 0.0335±0.0263 in IgE negative CD patients (N = 18), *P* = 0.046. This reduction was dependent on CD3+CD8+γδ T cells, 0.004±0.0038 in IgE positive CD patients *vs* 0.0093±0.0088 in IgE negative CD patients, *P* = 0.048; and CD3+CD56+γδ T cells, 0.0040±0.0076 in IgE positive CD patients *vs* 0.0054±0.0073 in IgE negative CD patients, *P* = 0.05. These results are consistent with the correlations showed in the Figure 2.

No significant differences were found in γδ T cells between patients with positive anti-*Encephalitozoon* IgE antibodies with respect to PCR results.

### Relations among positive anti-*Encephalitozoon* IgE antibodies in CD patients and IL-7 levels

IL-7 levels (pg/ml) were significantly lower in CD patients (13.1±8.7) compared to the control group (24.2±16.9), *P* = 0.021. In the clinical scenarios of CD, the new patient show 12.1±7.3, remission, 13.4±11.1 and active disease, 14.2±8.6 pg/ml.

In the group of CD, IgE anti-*Encephalitozoon* positive patients showed lower IL-7 levels (14.1±10.8) than the observed in the IgE negative patients, although significant differences were not observed.

Likewise, in the same group of CD patients, positive PCR results for microsporidia were related with significantly lower IL-7 levels (8.8±6.4) compared to negative PCR results (18.5±12.3), *P* = 0.021.

When correlations of IL-7 values with IgE anti-*Encephalitozoon* antibody levels were analyzed in patients with CD, an inverse relationship (Spearman rho: −225, no significant) was observed as in the case of γδ T cells (Fig. 2).

### Tissue staining

**Figure 4:**
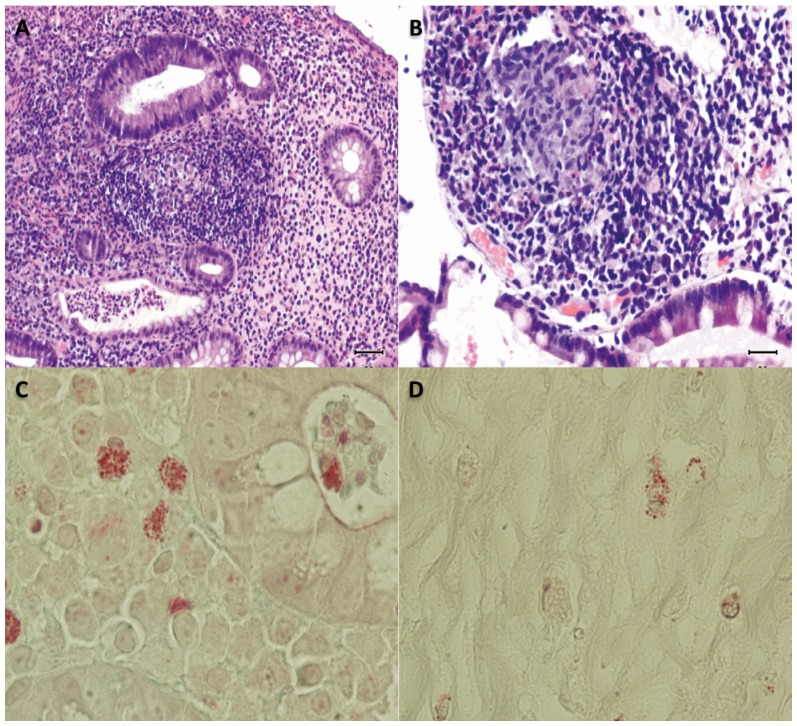
Tissue sampling of patient with CD. A, Chronic inflammation with lymphoid nodules in the lamina propria with cryptitis and crypt microabscesses in some. Hematoxylin-eosin stain x 200. B, Structure composed of granulomatous histiocytic added. Hematoxylin-eosin stain x 200. C, Staining of undetermined structures in the macrophages next to a crypt gland. Modified Trichrome stain x 1000. D, Staining of undetermined structures in the macrophages next to a crypt gland in healthy control. Modified Trichrome stain x 1000.

After the correlation observed for anti-*Encephalitozoon* IgE in CD patients, modified trichrome stain was carried out. All tissues analyzed from CD patients (N = 20) presented macrophages with undetermined structures inside (Figure 4) while only 20% of the controls (N = 3) presented these structures, OR = 7.6; CI95%, 2.7–22.0, *P*<0.001. Nevertheless the number of macrophages with these structures was higher in CD patients than in healthy controls (Figure 4C and D).

### Real time PCR

By means of the real time PCR, 30% of intestinal tissue samples of CD patients were infected with microsporidia (Table 1). From the patients with others diseases, 2/9 (22.2%) cases were also positive. These 2 patients were diagnosed as nonspecific ileitis and chronic diarrhea. Furthermore, all the patients of the control group were negative by real time PCR.

**Table 1:** Microsporidia positive and negative individuals by real time PCR.

		Crohn disease		Other disease*		Control	
		+	%	+	%	+	%
Phylum Microsporidia (undefined genera)		0	0	1	11.1	0	0
*Encephalitozoon*	*E. intestinalis/hellem*	1	5	1	11.1	0	0
	*E. cuniculi*	3	15	0	0	0	0
	Unknown species	1	5	0	0	0	0
*Enterocitozoon*	*E. bieneusi*	1	5	0	0	0	0
	**Total infected**	6	30	2	22.2	0	0
	Negative	14	70	7	77.8	15	100
	**Total**	20		9		15	

* Other diseases include: two chronic diarrheas, three nonspecific colitis, one diverticulitis, one fever of unknown origin, one irritable colon (not infected). Infected were: one nonspecific ileitis and one chronic diarrhea.

## Discussion

This paper describes for the first time a relationship between microsporidia and CD. In this work, we demonstrated higher IgE anti-*Encephalitozoon* antibody levels in CD patients with respect to the control group. With respect to IgE antibodies, it is interesting to note that Furuya et al [22] established three mouse monoclonal antibodies against *E. cuniculi* polar tube protein 1 (PTP1). All three monoclonal antibodies were IgE class, suggesting that, in addition to the highly immunogenic or antigenic nature, the PTP1 antigen may have the potential to induce specific IgE antibody production. These high IgE anti-*E. cuniculi* antibodies levels may be indicative of the presence of the microsporidia in the host causing a primary immune response.

The persistence of a pathogenic agent within macrophages can lead to typical granuloma formation in the context of a type IV hypersensitivity reaction in whose formation T lymphocytes, cytokines such as TNF, IFN-γ, as well as eosinophils and IgE are directly involved.

When correlations of anti-*Encephalitozoon* levels were studied with respect to lymphocyte subsets we observed that IgE anti-*Encephalitozoon* was negatively correlated with γδ T cells. This correlation was dependent on CD3+CD4+ and CD3+CD8+γδ T cells. Furthermore, we found a higher decrease of CD3+CD8+γδ T cells in IgE anti-*Encephalitozoon* positive CD patients. Moreover, CD patients with PCR positive result for microsporidia had significantly lower CD3+CD8+γδ T cell levels than negative PCR patients. It seems clear that CD8+T cells are essential in clearing microsporidia infection [27]. Likewise, Moretto et al [19] observed that T cells, due to their ability to produce cytokines, are important for the optimal priming of CD8+T cell immunity against *Encephalitozoon* infection.

It is interesting that, in healthy subjects, anti-*Encephalitozoon* IgE negatively correlated with αβ T cells, specifically CD4+αβ T cells. Likewise, it seems necessary to study this distinct action of T subsets against *Encephalitozoon* depending on the presence of absence of the disease since the prevalence of opportunistic intestinal parasitic infections including microsporidia is higher in patients with low CD4 cell counts [28].

Paradoxically, IgG anti-*Encephalitozoon* levels observed by us were much lower in the CD patients than in the healthy subjects. This could indicate that CD patients are not adequately immunized against microsporidia. The immune systems of these patients were unable to eradicate the pathogen. This would allow the pathogen to persist in the mucosal location, making the process chronic. This lack of IgG response could be explained by the deficit of T lymphocytes especially γδ T cells in CD patients that was previously demonstrated by us in an earlier work [1]. Furthermore, in the present study γδ T cells correlated with the levels of IgG anti-*Encephalitozoon* in patients with CD. By contrast, αβ T cells were not correlated with specific IgG levels, suggesting the involvement of γδ T cells, and specifically NK γδ T cells, in helping B cells to produce adaptive responses against *Encephalitozoon*.

Diagnosis of intestinal infection by microsporidia is traditionally carried out by Modified Trichrome stain, [29] followed by PCR techniques to determine the species using different samples, although fresh biopsies are preferred over paraffin-embedded tissues. It is well known that DNA extraction from these last samples yields a very small and degraded quantity of nucleid acid [30], [31]. The samples analyzed in our study were between four years old and a few months old, and therefore the integrity of DNA was very variable and unknown.

In our study, we have used a SYBR green real-time PCR for simultaneous detection and species identification of microsporidia previously described by Polley et al [26] for stool samples. Despite the fact that Velasquez et al [31] showed a better sensitivity than us in their assay, they analyzed only 11 samples described as positive by microscopy and 4 different PCR protocols were used to achieve 9/11 positive samples. None of the samples that we analyzed were suspicious of microsporidia infection and, moreover, the amount of tissue used was 5 times less than in the Velasquez et al [31] study. Nevertheless, we detected microsporidia in 8 samples. These make the Polley et al [26] protocol a very useful tool for microsporidia detection in old paraffin-embedded tissue samples.

Despite previous limitations, in our study we were able to diagnose the presence of microsporidia in 30% of cases of CD and in 2 (22.2%) in patients with other intestinal diseases. It is important to remark that one of these last patients presented a nonspecific ileitis that might be included in CD group due to its pathological analysis, despite the fact that he only presented one clinical flare-up. In the CD group we detected a case of *E. intestinalis/hellem*, a case of *E. bieneusi* and 3 cases of *E. cuniculi*. Due to the small number of positive samples studied, we can suspect of an association of *E. cuniculi* and CD, but more studies are needed to confirm this hypothesis or other alternatives ones, such as a better DNA conservation for this species over *E. intestinalis* and *E. bieneusi*. This is the first report of microsporidia in CD or any other autoimmune disease.

It is known since 1988 that there is an increase of antibodies to *Saccharomyces cerevisiae* in Crohn's disease [32], although these authors were not able to demonstrate the presence of the yeast in the tissues of CD patients. By means of the comparative study of kinome of both organisms (*E. cuniculi* and *S. cerevisiae*) it is known that they share ≈ 85% of their protein kinases [33]. It is therefore logical that cross-reactions would appear that would justify the increase of antibodies against *S. cerevisiae* in CD patients.

Previously, unidentified pathogens have been proposed as cause of CD in genetically susceptible individuals. Perhaps the genetic predisposition could be due to deficiencies of γδ T lymphocytes and IL-7 described by us recently in patients affected with CD [1]. Thus, these deficits would favour the colonization of the mucosa by these opportunistic organisms causing chronic disease. Works are in progress in order to further study this hypothesis.

In summary, our work demonstrated the presence of microsporidia in 30% of the tissues of patients with CD. In these patients, IgE anti-*Encephalitozoon* antibodies were increased while the specific IgG class was lower than in the control group. These results suggest that CD patients are a group at risk for microsporidiasis and, moreover that microsporidia may be involved in the etiology of CD.
